# Does epilepsy in multiplex autism pedigrees define a different subgroup in terms of clinical characteristics and genetic risk?

**DOI:** 10.1186/2040-2392-4-47

**Published:** 2013-12-01

**Authors:** Claire Amiet, Isabelle Gourfinkel-An, Claudine Laurent, Nicolas Bodeau, Bérengère Génin, Eric Leguern, Sylvie Tordjman, David Cohen

**Affiliations:** 1 Department of Child and Adolescent Psychiatry, Assistance Publique-Hôpitaux de Paris (AP-HP), Groupe Hospitalier Pitié-Salpêtrière, Université Pierre et Marie Curie, 47 bd de l’Hôpital, 75013 Paris, France; 2 IntegraGen, 5 rue Henri Desbruères, 91000 Évry, France; 3 Center of Epileptology, Reference Center for Rare Epilepsies, AP-HP, Groupe Hospitalier Pitié-Salpêtrière, 47 bd de l’Hôpital, 75013 Paris, France; 4 Department of Genetics, AP-HP, Groupe Hospitalier Pitié-Salpêtrière, Université Pierre et Marie Curie, 47 bd de l’Hôpital, 75013 Paris, France; 5 Centre de Recherche de l'Institut du Cerveau et de la Moelle épinière (CR-ICM), Université Pierre et Marie Curie/Institut national de la santé et de la recherche médicale (INSERM) Unité Mixte de Recherche (UMR) S975/Centre National de la Recherche Scientifique (CNRS) UMR 7225, Département Biotechnologies et Biothérapies, Groupe Hospitalier Pitié-Salpêtrière, 47 bd de l’Hôpital, 75013 Paris, France; 6 Department of Child and Adolescent Psychiatry, Groupe Hospitalier Guillaume Régnier, Université de Rennes, 108 Avenue Du General Leclerc, 35703 Rennes, France; 7 Institut des Systèmes Intelligents et Robotiques (ISIR), CNRS UMR 7222, Université Pierre et Marie Curie, 1 place Jussieu, 75005 Paris, France

**Keywords:** Autism, Multiplex pedigree, Epilepsy, Intellectual disability

## Abstract

**Background:**

Autism spectrum disorders (ASD) and epilepsy frequently occur together. Prevalence rates are variable, and have been attributed to age, gender, comorbidity, subtype of pervasive developmental disorder (PDD) and risk factors. Recent studies have suggested disparate clinical and genetic settings depending on simplex or multiplex autism. The aim of this study was to assess: 1) the prevalence of epilepsy in multiplex autism and its association with genetic and non-genetic risk factors of major effect, intellectual disability and gender; and 2) whether autism and epilepsy cosegregate within multiplex autism families.

**Methods:**

We extracted from the Autism Genetic Resource Exchange (AGRE) database (n = 3,818 children from 1,264 families) all families with relevant medical data (n = 664 children from 290 families). The sample included 478 children with ASD and 186 siblings without ASD. We analyzed the following variables: seizures, genetic and non-genetic risk factors, gender, and cognitive functioning as assessed by Raven’s Colored Progressive Matrices (RCPM) and Vineland Adaptive Behavior Scales (VABS).

**Results:**

The prevalence of epilepsy was 12.8% in cases with ASD and 2.2% in siblings without ASD (*P* <10^-5^). With each RCPM or VABS measure, the risk of epilepsy in multiplex autism was significantly associated with intellectual disability, but not with gender. Identified risk factors (genetic or non-genetic) of autism tended to be significantly associated with epilepsy (*P* = 0.052). When children with prematurity, pre- or perinatal insult, or cerebral palsy were excluded, a genetic risk factor was reported for 6/59 (10.2%) of children with epilepsy and 12/395 (3.0%) of children without epilepsy (*P* = 0.002). Finally, using a permutation test, there was significant evidence that the epilepsy phenotype co-segregated within families (*P* <10^-4^).

**Conclusions:**

Epilepsy in multiplex autism may define a different subgroup in terms of clinical characteristics and genetic risk.

## Background

Autism spectrum disorders (ASD) and epilepsy frequently occur together. Approximately 5% to 40% of individuals with autism have epilepsy [1], whereas the prevalence of epilepsy in the general population is about 0.5% to 1%. Conversely, 5% to 30% of individuals with epilepsy have ASD [2-5], whereas the prevalence of ASD in the general population is about 1/150; although recent studies reached even higher rates: 1/80 [6]. The variability of prevalence rates have been attributed to the heterogeneity of samples with respect to age, gender, comorbidity, subtype of ASD, intellectual disability and risk factors [7,8]. It may also result from the criteria used to diagnose epilepsy. Finally, the genetic background of autism may have a considerable impact on epilepsy rates in autism.

Recently, a meta-analysis of 24 reports on autism and epilepsy confirmed intellectual disability and female gender as major risk factors of epilepsy in autism [9]. The pooled prevalence of epilepsy was 21.4% in individuals with autism and intellectual disability versus 8% in individuals with autism without intellectual disability, and the more severe the intellectual disability, the more prevalent the epilepsy. With a male:female ratio of 2, the risk of epilepsy was found significantly higher for females [9]. However, this might reflect the known circumstance that females with autism tend to be more severely mentally disabled [10].

Autism and epilepsy are both complex and heterogeneous disorders. Recent literature reviews underline the important role of genetic factors in the etiology of ASD [11-13]. Rare chromosomal abnormalities (for example maternally inherited duplications of chromosome 15q11–q13) and single gene syndromes (for example fragile X, tuberous sclerosis) are important risk factors of ASD. More recently, individual genes of major effect have been identified by resequencing or array-based methods, but variants at these and other loci are present in no more than 1% to 2% of children with ASD [14]. Some of these genes with major effects have been associated with ASD, intellectual disability and/or seizures, suggesting that the three disorders may share common genetic risk factors (Table 1). Recent whole genome DNA microarray studies have identified submicroscopic duplications and deletions (copy number variations (CNVs) located in many loci and mostly including *de novo* events) in the same developmental disorders (ASD, intellectual disability, epilepsy), and in schizophrenia as well [15-17] (Table 2).

**Table 1:** Genes implicated in autism, epilepsy and/or intellectual disability

**Gene**	**Locus**	**Mutation**	**Transmission**	**Phenotype**	**Protein**	**Reference(s)**
*SCN1A*	2q24	Point mutation	*De novo*	ASD, E, ID	Na_v_1.1 (Na^+^ channel)	[18-20]
		Deletion	Dominant inheritance			
*SCN2A*	2q23–q24.3	Deletion	*De novo*	ASD, E, ID	Na_v_1.2 (Na^+^ channel)	[20-22]
		Point mutation	Inherited			
*SCN3A*	2q24	Deletion	*De novo*	E, ID	Na_v_1.3 (Na^+^ channel)	[23,24]
*SCN1B*	19q13.1	Point mutation	Dominant inheritance	E	β_1_ subunit (Na^+^ channel)	[25]
*KCNA1*	12p13	Point mutation	Dominant inheritance	E, ID	K_v_1.1 (K^+^ channel)	[26,27]
*KCNQ2*	20q13.3	Point mutation	Dominant inheritance	E, ID	K_V_7.2 (K^+^ channel)	[28,29]
		Deletion	*De novo*			
*KCNQ3*	8q24	Point mutation	Not known	E	K_V_7.3 (K^+^ channel)	[30]
*KCNMA1*	10q22	Point mutation	Dominant inheritance	ASD, E, ID	K_Ca_1.1 (K^+^ channel)	[31,32]
			*De novo*			
*CACNA1A*	19p13	Point mutation	*De novo*	E, ID	Ca_V_2.1 (Ca^2+^ channel)	[33]
			Dominant inheritance			
*GABRA1*	5q34–q35	Point mutation	Dominant inheritance	E	Α_1_ subunit (GABA_A_ receptor)	[34]
*GABRG2*	5q34	Point mutation	Dominant inheritance	E, ID	γ subunit (GABA_A_ receptor)	[35,36]
*CHRNA2*	8p21	Point mutation	Dominant inheritance	E	α_2_ subunit (nACh receptor)	[37]
*CHRNA4*	20q13.2–q13.3	Point mutation	Dominant inheritance	E, ID	α_4_ subunit (nACh receptor)	[38,39]
			*De novo*			
*CHRNB2*	1q21	Point mutation	Dominant inheritance	E	β_2_ subunit (nACh receptor)	[40]
			*De novo*			
*NLGN3*	Xq13.1	Point mutation	Inherited	ASD	Inhibitory synapse formation	[41]
*NLGN4*	Xp22.31	Point mutation	Inherited	ASD, ID	Synapse formation	[41,42]
		Deletion				
*CDH8*	16q21	Deletion	Inherited	ASD	Synapse formation	[43]
*PCDH10*	4q28.3	Deletion	Inherited	ASD	Synapse formation	[44]
*PCDH19*	Xq22	Deletion	*De novo*	E, ID	Synapse formation	[45]
		Point mutation	Inherited			
*NRXN1*	2p16.3	Deletion	Recessive inheritance	ASD, E, ID, SCZ	Synapse formation	[46-48]
		Point mutation	*De novo*			
*CNTNAP2*	7q35	Deletion	Recessive inheritance	ASD, E, ID, SCZ	Synapse formation	[49-51]
		Point mutation	*De novo*			
*SHANK2*	11q13.4	Deletion	*De novo*	ASD, ID	Synapse scaffolding	[52]
		Point mutation	Inherited			
*SHANK3*	22q13.3	Deletion	*De novo*	ASD, ID, SCZ	Synapse scaffolding	[53,54]
		Point mutation	Inherited			
*SYNGAP1*	6p21.3	Point mutation	*De novo*	ASD, E, ID	Synapse RasGAP	[55,56]
		Deletion	Inherited, *de novo*			
*CDKL5*	Xp22	Point mutation	*De novo*	E, ID	Cyclin-dependent kinase-like 5	[57,58]
		Deletion	Inherited			
*ARX*	Xp22.13	Duplication	Inherited	ASD, E, ID	Aristaless-related homeobox protein	[59,60]
			*De novo*			
*ATP1A2*	1q21–23	Point mutation	Dominant inheritance	E, ID	Sodium-potassium ATPase	[61,62]
*SLC2A1*	1p35–p31.1	Deletion	Dominant inheritance	E, ID	GLUT1	[63]
		Point mutation	*De novo*			
			Recessive inheritance			
*STXBP1*	9q34.1	Point mutation	Inherited	E, ID	Syntaxin-binding protein	[64]
			*De novo*			

Adapted from Betancur [15] and Amiet [65]. ASD, autism spectrum disorders; E, epilepsy; GABA, γ-aminobutyric acid; GLUT1, glucose transporter type 1; ID, intellectual disability; nACh, nicotinic acetylcholine; RasGAP, Ras GTPase activating protein; SCZ, schizophrenia.

**Table 2:** Copy number variations (CNVs) associated with autism spectrum disorders (ASD), epilepsy, intellectual disability and schizophrenia

**Cytoband**	**Maximum coordinates**	**Phenotype**	**Deletion/duplication**	**References**
1q21.1	144.9–146.2	ASD, E, ID, SCZ	Deletion/duplication	[16,66-75]
3q29	197.1–198.9	ASD, E, ID, SCZ	Deletion/duplication	[16,74-81]
7q11.23	71.9–74.2	ASD, E, ID, SCZ	Deletion/duplication	[69,75,82-89]
15q11.2–13.1	21.1–26.2	ASD, E, ID, SCZ	Duplication	[68,69,72,83,90,91]
15q13.3	28.2–30.7	ASD, E, ID, SCZ	Deletion/duplication	[16,68-72,75,83,92-100]
16p11.2	29.4–30.1	ASD, E, ID, SCZ	Duplication	[16,68,74,75,83,101-107]
16p11.2	29.4–30.1	ASD, E, ID	Deletion	[68,83,92,101,102,104-110]
16p13.11	14.6–18.7	ASD, E, ID, SCZ	Deletion/duplication	[68,69,72,73,81,90,92,111-114]
17q12	31.5–33.1	ASD, E, ID, SCZ	Deletion/duplication	[73,81,115-117]
22q11.2	16.9–20.6	ASD, E, ID, SCZ	Deletion	[16,68,69,71,72,80,92,101],[118,119]

Adapted from Betancur [15], Johnson and Shorvon [17], and Levinson *et al.*[120]. ASD, autism spectrum disorders; CNV, copy number variation; E, epilepsy; ID, intellectual disability; SCZ, schizophrenia.

However, all together, an identified genetic etiology is estimated to account for 10% to 20% of ASD cases [15]. Therefore, in the majority of epilepsy cases, most autism appears to be a multifactorial hereditary disorder, depending on polygenic heredity and environmental factors Environmental factors associated with autism are mainly pre-, peri- and postnatal, such as *in utero* exposure to teratogenic medications (for example thalidomide, valproate) [121], prematurity, neonatal encephalopathy and hyperbilirubinemia [122].

Most of the samples studied in autism are composed of autistic individuals without information about whether autism affects only one individual in the family (simplex autism) or multiple siblings in the family (multiplex autism). This is a major issue since higher genetic risk may be hypothesized in multiplex autism. Some recent studies have suggested disparate clinical and genetic settings depending on simplex or multiplex autism. More *de novo* rare mutations in genes of major effect have been associated with simplex autism [108], while an interactive effect of multiple common susceptibility alleles have long been hypothesized as risk factors of familial autistic syndromes [123,124]. Whether some clinical variables may help to delineate more homogeneous subgroups in multiplex autism remains an open question. Regarding epilepsy and ASD, only one study specified that part of the sample included individuals with multiplex autism [125]. However, there was no separate analysis of these two groups except to observe that a similar incidence of electroencephalogram (EEG) abnormalities was found. Banach *et al*. examined gender differences and intellectual quotients (IQs), and found no difference in non-verbal IQ between females and males in multiplex families, while females had lower non-verbal IQ scores than males in simplex families [126]. However, the prevalence of major effect genetic risk factors as a function of epilepsy, intellectual disability and gender in multiplex families remains unknown.

The aims of the current study are: 1) to assess the prevalence of seizures in multiplex autism and compare it to the prevalence of siblings without ASD. Since autism and epilepsy share common genetic risk factors, we hypothesize a higher risk of epilepsy in individuals with autism than siblings without ASD; 2) to compare the prevalence of major effect genetic risk factors in multiplex autism with and without epilepsy. We hypothesize the group with epilepsy to have an increased risk; 3) to assess whether autism and epilepsy co-segregate within multiplex autism families; and 4) to assess whether higher prevalence of epilepsy in multiplex autism is mediated by intellectual disability and gender as it has been shown in the literature [9].

## Methods

### Participants

Data were ascertained from all families participating in the Autism Genetic Resource Exchange (AGRE) in September 2011. Families with two or more members diagnosed with autism, pervasive developmental disorder-not otherwise specified (PDD-NOS) or Asperger syndrome may enroll in the AGRE program. Families register to the program then AGRE recruitment staff follow-up with families to confirm eligibility and complete the intake process. Families were excluded from the AGRE program if they had participated in another genetic study, a confirmed neurogenetic disorder, a pre- or perinatal injury, prematurity defined as delivery before 35 weeks of gestational age for single births or before 33 weeks of gestational age for twin births, a known chromosomal abnormality, or an abnormal neurological examination.

The diagnosis of autism was made using the standard Autism Diagnostic Interview-Revised (ADI-R) [127] with three affected status categories based on ADI-R results: autism, not quite autism (NQA) and broad spectrum. In the current report, we fused the last two categories as PDD-NOS. To meet the criteria for autism, the individual must exceed the ADI-R cutoff for autism in all domains. Children meeting the following criteria were classified as PDD-NOS: 1a) must meet cutoff scores for autism on the three core domains (social, communication, behavior) but not on the age of onset domain, or 1b) must be no more than one point below the cutoff score on any of the three core domains, while also meeting the cutoff for the age of onset domain; and 2) must meet one or more of the following: a) show a severe deficit in at least one domain, b) show more moderate deficits in at least two domains, and/or c) show only minimal deficits in all three domains.

The AGRE database consisted of 3,819 children from 1,264 families. We excluded the 3,150 children (1,837 children with autistic disorder, 226 children with PDD-NOS and 1,087 children without autism) whose clinical data gave no information about the presence or absence of epileptic seizure. We excluded five more individuals who were reported by their family with a diagnosis of Asperger syndrome without any ADI-R available. We included the 664 children from 290 families (417 children with autistic disorder, 61 children with PDD-NOS and 186 children without autism) whose characteristics are discussed below. We found no significant differences between excluded and included individuals for age at ADI-R clinical assessment (mean (± SD)) (8.5 years (± 4.9) versus 8.1 years (± 4.7), respectively; *P* = 0.098), gender (68.1% versus 69.7% of boys, respectively; *P* = 0.43) and Raven’s nonverbal IQ (100.2 (± 19.3) versus 99.8 (± 18), respectively; *P* = 0.76). We found a significant difference in the percentage of autistic disorder, PDD-NOS and unaffected children (*P* = 0.002), but the difference accounted for a higher percentage of unaffected children in the excluded sample (34.6% versus 28.0%). In contrast with Raven’s nonverbal IQ, we found a significant difference for Vineland composite standard score (62.6 (± 21.6) versus 54.4 (± 20.4), respectively; *P* <10^-10^).

### Measures

#### Data relevant to epilepsy

Most of the AGRE families had an extensive evaluation by pediatricians, psychiatrists and/or neurodevelopmental specialists in order to obtain medical and family history information. Data relevant to epilepsy were extracted from probands, and unaffected children’s medical history files were provided by the AGRE database. These data were collected through a standardized method by AGRE and consist of the occurrence or not of afebrile seizures or remote febrile seizures, the age of onset, and the number of seizures and their type. After extraction from the AGRE database, data were reviewed by a neurologist and three groups were formed: epilepsy, remote febrile seizures (meaning febrile seizures with an identified antecedent classified as a risk factor for epilepsy) and only one seizure. Epilepsy was defined as two or more afebrile seizures.

#### Genetic and non-genetic risk factors of autism

For each of the 478 included individuals with ASD, we determined whether there were known genetic and non-genetic risk factors of autism. Despite the exclusionary criteria described above, the database flags: 1) possible syndromic autism based on clinical (significant dysmorphology, abnormal neurologic exams) and morphologic criteria (abnormal imaging or medical tests); and 2) pre- or perinatal injuries and prematurity (defined as delivery before 35 weeks of gestational age for single births and before 33 weeks of gestational age for twin births). Also noted were: comorbid medical or psychiatric disorders and genetic abnormalities identified by clinical genetic investigation [128]. Most of the families of the AGRE dataset were screened for fragile X syndrome using PCR technique. About half of the AGRE families had been karyotyped, and 228 (47.7%) were analyzed for small nuclear ribonucleoprotein polypeptide N (SNRPN) duplication at 15q12 and 393 for telomere analyses for small-scale deletions and duplications. A sample of 943 families was genotyped using the HumanHap550 BeadChip (Illumina, San Diego, CA, USA) [129] and a sample of 777 families was genotyped using the 5.0 Chip (Affymetrix, Santa Clara, CA, USA) [130]. The details of these screenings are available on the AGRE website (http://www.agre.org).

Of the 664 children included in this analysis, 35 (5.3%) children from 33 families (31 children with autistic disorder, two children with PDD-NOS and two children without autism) were flagged as having strong risk factors for both autism and seizures: pre- or perinatal insult, prematurity before 35 weeks of gestational age (19 children), chromosomal abnormality (eight children with a known trisomy, translocation or inversion, and one child with significant dysmorphology), or an abnormal neurological or cerebral imaging examination (seven children). We found no significant differences between these children and their siblings and the children with no flag for age on ADI-R clinical assessment (*P* = 024), gender (*P* = 0.42), mean Raven’s non-verbal IQ (*P* = 0.17), mean Vineland composite standard score (*P* = 0.80), or the percentage of autistic disorder, PDD-NOS and unaffected children (*P* = 0.17).

#### Raven’s Colored Progressive Matrices (RCPM)

Non-verbal intellectual capacity was measured by Raven’s Colored Progressive Matrices (RCPM) [131]. The RCPM is a standardized test designed to measure non-verbal intellectual capacity in children from the age of 5 years. Scores on the RCPM correlate highly with scores on tests of general intellectual capacity [132]. The RCPM consist of 36 items, presented in three sets of 12, which become progressively more difficult. Each item contains a pattern problem with one part removed and six pictured potential inserts, one of which contains the correct pattern. The number of correct answers is transformed to a non-verbal IQ score based on age-dependent normative data.

#### Vineland Adaptive Behavior Scales (VABS)

The adaptive functioning level was assessed by the Vineland Adaptive Behaviors Scales (VABS). The VABS is a semi-structured parental interview that evaluates adaptive functioning in four domains: communication, daily living skills, socialization and motor skills. Age equivalent scores and standard scores are provided for each domain. Scores across domains can be combined to create an overall adaptive behavior composite standard score. Depending on the time of inclusion in AGRE, one of the two forms of the VABS was used: the survey form [133] and the second edition of the VABS (Vineland II) [134]. For the purposes of the current study, the overall adaptive behavior composite standard score was used.

#### Statistical analysis

R software, version 2.12.2, was used for the statistical analysis. First, we compared affected individuals with individuals without epilepsy. Fisher’s exact test was used for analysis of the association between the qualitative variables. A Student’s *t*-test was used for the analysis of the association between the quantitative variables and the group variables. Second, we tested whether epilepsy co-segregated within families. To do so, from the 290 families, we first extracted the 179 families in which pairs of two affected siblings had a complete dataset for epilepsy, since permutation tests cannot be applied on missing data. Each sib-pair was designated either concordant or discordant for epilepsy status. In each of the 20,000 permutations, the individuals with epilepsy were randomly assigned among the 179 families, and the resulting number of concordant sib-pairs was recorded. The *P* value is the fraction of permutation in which the number of concordant sib-pairs exceeded the number in the observed data. Third, admixture analysis was used to determine the best-fit model for the age at onset of seizure in ASD. Given the literature, we hypothesized a model with two subgroups, including one with seizure onset before the age of 5 years and another with seizure onset close to adolescence. Finally, a multivariate analysis (logistic regression) was performed to assess which variables contribute to the risk of seizures.

To assess whether the inclusion of the 35 children from 33 families who were flagged with strong risk factors for autism and seizures biased our results, we performed secondary analyses excluding all families with such children. These analyses were performed on 586 children from 257 families who had medical data about presence or absence of epilepsy: 362 (61.8%) children with autistic disorder, 53 (9%) children with PDD-NOS and 171 (29.2%) children without autism.

## Results

Primary analyses were performed on 664 children from 290 families who had informative data about presence or absence of epilepsy: 417 (62.8%) children with autistic disorder, 61 (9.2%) children with PDD-NOS and 186 (28%) children without autism (Table 3). Secondary analyses were performed on 586 children from 257 families after exclusion of children with strong risk factors for autism and seizures.

**Table 3:** Sample characteristics and prevalence of epilepsy according to clinical status

**Characteristics**	**Autistic disorder**	**PDD-NOS**	**Total (ASD)**	**Siblings without autism**
Total (n)	417	61	478	186
Gender (male/female)	334/83	48/13	382/96	81/105
Mean age (years)	9.33 (± 5.02)	8.67 (± 5.01)	9.24 (± 5.02)	10.56 (± 6.59)
Epilepsy	56 (13.4%)	5 (8.2%)	61 (12.8%)	4 (2.2%)
Remote febrile seizures	16 (3.8%)	6 (9.8%)	22 (4.6%)	5 (2.7%)
One afebrile seizure	14 (3.4%)	0 (0%)	14 (2.9%)	1 (0.5%)
Risk factors for ASD	39 (9.6%)	3 (5.4%)	42 (8.8%)	0
Risk factors for ASD and comorbid seizures	10 (2.5%)	0	10 (2.2%)	0

Performed on 664 children and 290 families. ASD, autism spectrum disorders; PDD-NOS, pervasive developmental disorder-not otherwise specified.

### Prevalence and type of epilepsy

The prevalence of epilepsy was 13.4% (56/417) among cases with an autistic disorder, 8.2% (5/61) among cases with PDD-NOS and 2.2% (4/186) among siblings without autism (*P* <10^-4^). The risk of epilepsy was sevenfold higher for children with ASD (autistic disorder or PDD-NOS) when compared with their siblings without ASD (odds ratio (OR) = 0.15; 95% confidence interval (CI): 0 to 0.4; *P* <10^-5^). The prevalence of remote febrile seizures was 4.6% (22/478) in cases with an ASD and 2.7% (5/186) in siblings without ASD (OR = 1.75; 95% CI: 0.63 to 5.99; *P* = 0.38). The prevalence of one afebrile seizure was 14/478 (2.9%) in cases with an ASD and 0.5% (1/186) in siblings without ASD (OR = 5.57; 95% CI: 0.84 to 237.1; *P* = 0.08). Secondary analyses excluding children with strong risk factors for autism and seizures yielded similar results: the prevalence of epilepsy was 12.2% (44/362) in cases with an autistic disorder, 5.7% (3/53) in cases with PDD-NOS and 2.3% (4/171) in siblings without autism (*P* = 0.0002).

**Figure 1:**
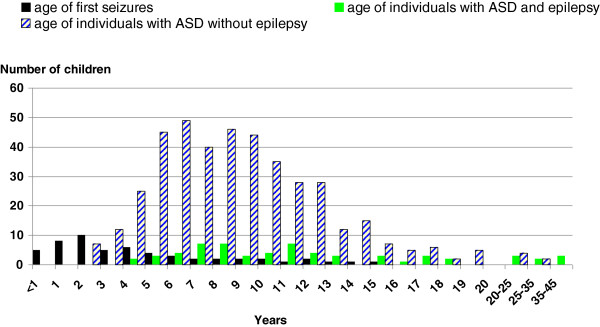
**Distribution of age of first seizure, age of individuals with ASD and epilepsy, and age of individuals with ASD without epilepsy.** Age of first seizure (n = 55, black), age of individuals with ASD and epilepsy (n = 61, green or grey), and age of individuals with ASD without epilepsy (n = 417, white and blue). ASD, autism spectrum disorders.

**Figure 2:**
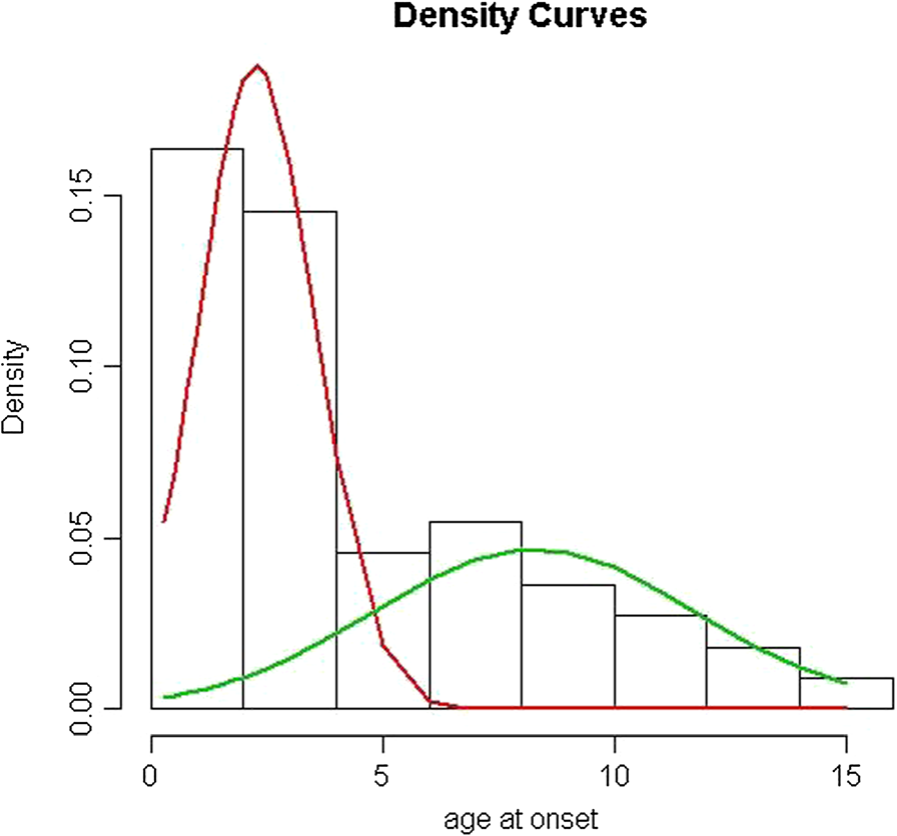
**Theoretical distribution of ages of onset of seizures for 55 individuals with comorbid multiplex autism with epilepsy.** Distribution shows two subgroups with early onset of seizures (mean = 2.3 years (± 1.3)) and late onset of seizures (mean = 8.3 years (± 3.5)). A parametric bootstrap with B = 500 replications of the likelihood ratio statistic was performed for testing the null hypothesis of a one-component fit versus the alternative hypothesis of a two-component fit. The result was significant (*P* = 0) and therefore the model with two components was adequate.

The mean age of children with ASD was 9.2 years (± 5.0) at the time of their clinical assessment. Children with ASD and epilepsy were significantly older (mean age: 12.8 years ± 8.6) than children with ASD without epilepsy (mean age: 8.7 years ± 4.0) (*P* <10^-8^). Mean age of the first seizure was 4.7 years (± 3.8). Figure 1 summarizes the distribution of seizure onset according to age. Given the 4-year older age of children with epilepsy, the distribution of age of individuals with ASD without epilepsy, and of individuals with ASD and epilepsy, is also given. As several reports showed that there may be distinct ages at onset of subgroups among individuals with epilepsy, we conducted an admixture analysis to test whether the observed distribution for age at onset in individuals with ASD and seizure (n = 55) was a mixture of Gaussian distributions. A parametric bootstrap with B = 500 replications of the likelihood ratio statistic was performed for testing the null hypothesis of a one-component fit versus the alternative hypothesis of a two-component fit. The result was significant (*P* <0.001) and therefore the model with two components was adequate. The mean ages estimated in this model were 2.3 years (± 1.3) and 8.3 years (± 3.5) (Figure 2). All types of seizures were reported in children with autism and epilepsy (n = 61): 12 (19.7%) children had generalized seizures, 17 (27.9%) children had absences, 14 (23.0%) children had complex partial seizures, 11 (18.0%) children had multiple seizure types, and the type of seizure was unknown for seven (11.5%) children.

Secondary analyses excluding children with strong risk factors for autism and seizures yielded similar results: mean age of ASD children = 9.1 years (± 4.7); mean age of ASD and epilepsy children = 12.4 years (± 7.0) versus mean age of ASD children without epilepsy = 8.7 years (± 4.1) (*P* <10^-6^); mean age at the first seizures = 5.2 years (± 4.1); similar proportions of generalized seizures (21.3%), absences (27.7%), complex partial seizures (23.4%), multiple seizures types (19.1%), and unknown (8.5%).

### Co-segregation analyses and prevalence of genetic and non-genetic risk factors as a function of epilepsy

Of the 478 children with ASD, 464 (97.1%) children underwent genetic testing (karyotyping and/or array comparative genomic hybridization (aCGH), and/or genome-wide association (GWA)). A total of 42 (8.8%) children had a known possible genetic or non-genetic risk factor for autism (39 children with autistic disorder, three children with PDD-NOS; Table 4). In decreasing frequency, we found prematurity and/or pre- or perinatal insult (n = 19), chromosomal abnormality (n = 14), abnormal cerebral imaging or significant developmental abnormality (n = 3), comorbid medical disease associated with ASD (n = 4), and cerebral palsy (n = 2). An epilepsy was reported for ten children (all had autistic disorder). Genetic or non-genetic risk factors of autism tended to be significantly associated with epilepsy (OR = 2.25; 95% CI: 0.93 to 5.04; *P* = 0.052). When children with prematurity, pre- or perinatal insult, or cerebral palsy were excluded, a known or possible genetic risk factor was reported for 10.2% (6/59) of children with epilepsy and 3.0% (12/395) of children without epilepsy (OR = 3.6; 95% CI: 1.06 to 10.9; *P* = 0.02).

**Table 4:** Risk factors of autism in the AGRE sample according to epilepsy

**Risk factors**		**Total (n)**	**Epilepsy (n)**	**No epilepsy (n)**
Total of individuals		478	63	415
Chromosomal abnormality or genetic condition		**14 (2.92%)**	**4 (6.35%)**	**10 (2.4%)**
	16p11.2 deletion/duplication	4	2	2
	15q11–13 duplication	3	1	2
	22q11.21 duplication	1	0	1
	22q13.33 duplication	1	0	1
	t(2;9)(p13;q34.3)	1	1	0
	Mosaic t(3;16)	1	0	1
	Mosaic t(3;14)	1	0	1
	Trisomy 21	1	0	1
	Mosaic trisomy 12	1	0	1
Prematurity^a^/pre- or perinatal insult		**19 (3.98%)**	**3 (4.76%)**	**16 (3.9%)**
Cerebral palsy		**2 (0.42%)**	**0**	**2 (0.48%)**
Abnormal cerebral imaging		**3 (0.63%)**	**2 (3.17%)**	**1 (0.24%)**
	Agenesis of corpus callosum	1	1	0
	Left frontal damage	1	1	0
	Not given	1	0	1
Other significant abnormality		**4 (0.84%)**	**1 (1.59%)**	**3 (0.72%)**
	Significant dysmorphology	2	0	2
	Mitochondrial disorder, mild tonsillectomy (premature puberty)	1	1	0
	Cranial nerve paralysis (congenital versus traumatic)	1	0	1
Total of individuals with abnormalities^b^		42 (8.8%)	10 (15.9%)	32 (7.71%)

^a^Defined as delivery before 35 weeks of gestational age for single births or before 33 weeks of gestational age for twin births; ^b^*P* = 0.052. AGRE, Autism Genetic Resource Exchange.

Finally, there was significant evidence that epilepsy co-segregated within autism sib-pairs. Indeed, among the 179 families with sib-pairs affected by ASD, we found 154 sib-pairs concordant (meaning both siblings with ASD had epilepsy or did not have epilepsy) for associated epilepsy, while 25 sib-pairs were discordant (permutation: *P* <10^-4^). Secondary analyses excluding children with strong risk factors for autism and seizures yielded similar results: among the 155 families with sib-pairs affected by ASD, we found 132 sib-pairs concordant for associated epilepsy, while 23 sib-pairs were discordant (permutation test: *P* = 0.0049). Table 5 details the 11 AGRE families with two children with ASD and comorbid epilepsy, showing clinical status, type of seizure and known risk factors in all siblings.

**Table 5:** **AGRE families with two children with ASD and comorbid epilepsy (n = 11): clinical status, type of seizures and risk factors in all siblings**^**a**^

**Family**	**Gender**	**Autism diagnosis**	**Type of seizure**^**b**^	**Risk factor of ASD and/or epilepsy**
AU0025	M	Autism	Partial	Mitochondrial disorder, mild tonsillectomy (premature puberty)
	M	Autism	Multiple	No risk reported
	F	Unaffected	No seizure	Unknown
AU0051	M	Autism	Absence	No risk reported
	M	Autism	Absence	Agenesis of corpus callosum
AU0123	F	Autism	Partial	Prematurity^c^/pre- or perinatal insult
	M	Autism	Multiple	Unknown
	F	Unaffected	No seizure	Unknown
AU0176	F	Autism	Partial	No risk reported
	M	Autism	Partial	No risk reported
	M	Unaffected	No seizure	Unknown
	F	Unaffected	No seizure	Unknown
AU0275	M	Autism	Multiple	No risk reported
	M	Autism	Not indicated	Unknown
	F	Unaffected	No seizure	Unknown
	F	Unaffected	No seizure	Unknown
AU0461	M	Autism	Generalized	No risk reported
	M	Autism	Generalized	Unknown
AU0548	F	Autism	Absence	No risk reported
	M	Autism	Not indicated	No risk reported
	F	Unaffected	No seizure	Unknown
AU0731	M	Autism	Generalized	Prematurity^c^/pre- or perinatal insult
	M	PDD-NOS	Not indicated	Unknown
AU0765	M	Autism	Absence	Unknown
	F	PDD-NOS	Not indicated	t(2;9)(p13;q34.3)
	M	Unaffected	No seizure	Unknown
AU0922	M	Autism	Partial	No risk reported
	F	Autism	Partial	Unknown
AU1208	M	Autism	Multiple	15q11–13 duplication
	M	Autism	Absence	Unknown

^a^From the 154 families included in the co-segregation analysis, the families with ASD children and without epilepsy are not detailed in this table; ^b^not indicated means that the type of seizures was not given in the AGRE database, although the patient exhibits epilepsy; ^c^defined as delivery before 35 weeks of gestational age for single births or before 33 weeks of gestational age for twin births. AGRE, Autism Genetic Resource Exchange; ASD, autism spectrum disorders; PDD-NOS, pervasive developmental disorder-not otherwise specified.

### Epilepsy and non-verbal IQ/adaptive level

Given the heterogeneity of general cognitive function in ASD, we used two measures to assess whether epilepsy was associated with intellectual disability in multiplex autism. An assessment with the RCPM was applied to 438 children with ASD (381 children with an autistic disorder and 57 children with PDD-NOS). One hundred forty-three children were not testable and ten children scored above the highest possible age-specific non-verbal IQ, without numeric scores available. The mean non-verbal IQ of children with ASD (n = 285) was 99.6 (± 18.0). Stratification of cases into two categories: IQ <70 (n = 17) and IQ ≥70 (n = 278) showed a fivefold increased risk of epilepsy for children with an IQ <70 (7/17; 41.2%) comparing children with an IQ ≥70 (31/278; 11.2%) (OR = 5.5; 95% CI: 1.7 to 17.5; *P* = 0.005).

An assessment with VABS was performed in 386 children with ASD (336 children with an autistic disorder and 50 children with PDD-NOS). The mean overall adaptive behavior composite standard score of children with ASD was 54.0 (± 20.2). Stratification of cases into two categories: composite standard score <70 (n = 294) and composite standard score ≥70 (n = 92) showed a threefold increased risk of epilepsy for children with a composite standard score <70 (45/294 (15.3%)) comparing children with a composite standard score ≥70 (5/92 (5.4%) (OR = 3.1; 95% CI: 1.2 to 10.5; *P* = 0.015). Figure 1 depicts the frequency of comorbid epilepsy in individuals with ASD as a function of composite standard score. The more severe the impairment of adaptive level, the more prevalent the epilepsy (*P* = 0.002).

### Epilepsy and gender

Among the 96 females with a diagnosis of ASD (autistic disorder or PDD-NOS), 16.7% (n = 16/96) had epilepsy. For the 382 males with ASD, 11.8% (45/382) had epilepsy. The male:female ratio was 4.2 for the children with ASD without epilepsy, and 2.9 for the children with ASD and with epilepsy. However, the difference was not statistically significant (OR = 0.7; 95% CI: 0.3 to 1.3; *P* = 0.268). Considering all the children with ASD regardless of their epileptic status, the male:female ratio was not significantly different when the sample was stratified into two groups (normal intelligence versus intellectual disability) based on the non-verbal IQ (RCPM: ≥70, n = 278; <70, n = 17) (male:female ratio = 3.3 versus 4.5, respectively) (OR = 0.7; 95% CI: 0.2 to 3.2; *P* = 0.788) or the adaptive level (VABS: ≥70, n = 92; <70, n = 294) (male:female ratio = 3.4 versus 3.9, respectively) (OR = 1.2; 95% CI: 0.6 to 2.1; *P* = 0.664). Furthermore, there was no difference when the sample was stratified into four groups based on the adaptive level (<40, 40 to 54, 55 to 69, ≥70; *P* = 0.591).

### Multivariate analysis

To confirm univariate variables associated with the risk of seizures, we used a logistic regression. ASD (estimate = 2.49, *P* = 1.1 10^-5^; OR = 12; 95% CI: 4.46 to 43.1), age (estimate = 0.11, *P* = 1.1 10^-6^; OR = 1.15; 95% CI: 1.07 to 1.17) and gender (male: estimate = -0.61, *P* = 0.049; OR = 0.54; 95% CI: 0.3 to 1.013) were associated with the risk of seizure. In ASD individuals only, gender was not significantly associated, whereas age and intellectual disability were significantly associated, whether intellectual disability was measured with VABS (estimate = 1.15, *P* = 0.02; OR = 3.2; 95% CI: 1.3 to 9.6) or with RCPM (estimate = 1.57, *P* = 0.005; OR = 4.85; 95% CI: 1.53 to 14.56).

## Discussion

Epilepsy in autism has been a subject of increasing interest, and reported prevalence rates of seizures in this condition vary from 5% to 40% [1]. The variability of rates have been attributed to the heterogeneity of samples with respect to age, gender, comorbidity, subtype of pervasive developmental disorder (PDD), intellectual disability and risk factors [9]. But most of the studies have been conducted in samples with simplex autism; for example the prevalence found in the Simons Simplex Collection (a large multisite phenotypic and DNA collection that includes families (n = 2,644) in which there is only one child (aged 4 to 17 years) with ASD) is 2.4% [135]. In multiplex families, we found that 13.4% of children with autism and 8.2% of children with PDD-NOS had epilepsy. These results are in the range of prevalence rates described in the literature. In these multiplex families, we found that 2.2% of the siblings without autism had epilepsy. This prevalence rate is high compared to the general population where 0.7% to 0.8% of children up to the age of 15 years have repeated seizures [136]. Moreover, the permutation tests performed in this study show significant evidence that epilepsy phenotypes co-segregated within families.

Studies suggest that there are two peaks of onset of seizures in autism, one in early childhood (before the age of 5 years old), and one near adolescence [137]. In the sample studied here, the admixture analysis confirms this two-peak model: 62% of the children had their first seizures before the age of 5 years. Mean age of onset for the second peak is in pre/early adolescence as only 9% of the children had their first seizure after the age of 12 years. However, with a mean age of 9.2 years, the children studied are relatively young and many of them had not yet reached adolescence. Therefore, it is likely that our estimate of the age of onset for the later peak may be spuriously low as we cannot rule out that epilepsy could begin later for some individuals. Unfortunately, conducting a subanalysis with individuals aged 16 years and older was not possible given the sample size.

**Figure 3:**
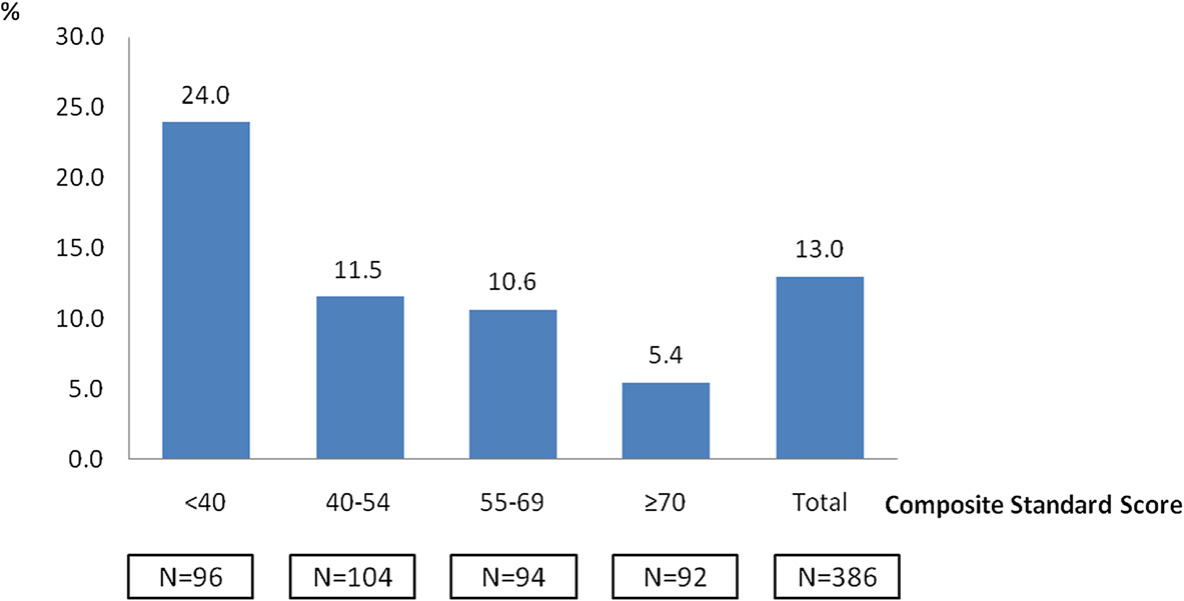
Frequencies of comorbid epilepsy in children (n = 386) with autism from multiplex families as function of the Vineland Adaptive Behavior Scales (VABS) composite standard score.

The risk of epilepsy in individuals with simplex autism was significantly associated with intellectual disability and gender. In a meta-analysis of 17 studies, the pooled prevalence of epilepsy was 21.5% in individuals with autism and intellectual disability, against 8% in individuals with autism and normal intelligence [9]. Of note, the increased risk with gender in simplex autism may reflect the known circumstance that females with autism tend to have more severe intellectual disability: the more severe the intellectual disability, the lower the male:female ratio [10]. In the present study, the respective prevalence rates of epilepsy was 41.2% in individuals with autism and intellectual disability, against 11.1% in individuals with autism and normal intelligence (*P* = 0.005), and the more the adaptive behavior level was impaired, the higher the prevalence of epilepsy (Figure 3). However, there was no increase of epilepsy linked to gender in individuals with ASD. Furthermore, no significant difference in IQ among males and females was found in the AGRE sample. A similar result was suggested in previous reports of multiplex families [126,138].

Identification or strong suspicion of a risk factor for autism (genetic or not) tended to increase the prevalence of epilepsy. The non-genetic risk factors identified were mainly perinatal fetal distress, the implication of which for autism, epilepsy and intellectual disability is well known. The prevalence of epilepsy remained significantly higher even when only identified or suspected genetic risk factors were considered. Genetic risk factors may be more often implicated when autism and epilepsy are associated [139]. This is also the case in multiplex families.

Interpretation of the informative families presented in Table 5 requires consideration of the AGRE database limitations, which include: excluding individuals with major perinatal risk factors or with known genetic abnormalities (for example fragile X syndrome) (but some siblings with such risk factors may have been included); and in many individuals, data are unknown or missing (for example not all individuals underwent CNV search; see Methods). Also, large family histories including epilepsy and ASD statuses of all members were not available. Careful scrutiny of Table 5 shows that several combinations of risk can lead to a multiplex pedigree: 1) the existence of a common risk factor for epilepsy and autism leading to the suggestion of screening for known genetic risk factors of epilepsy in AGRE families AU25, AU51, AU176, AU275, AU461, AU548 and AU922; and 2) the co-occurrence of two different risk factors (genetic and non-genetic ones) leading to a false ‘multiplex pedigree’, such as in families AU731, AU123, AU765 and AU1208; and 3) combinations of major and minor risk factors as it cannot be excluded that a) in some families interpretation of genotype/phenotype relationship may be particularly complex (for example AU25, AU51) and b) the same ASD genetic risk factor may be responsible for increased risk of prematurity (for example AU731).

In terms of pathophysiological mechanisms, several hypotheses have been proposed to explain the comorbidity between autism and epilepsy: 1) accidental co-occurrence given the high frequency of both conditions; 2) altered internal organization of minicolumns in the cortex of autistic individuals may be associated with a defect in inhibitory local circuit projection, and the defect in γ-aminobutyric acid (GABA)ergic fibers may correlate with the increased prevalence of seizures in autism [140,141]; 3) common genetic or neurodevelopmental risk factors; and 4) epilepsy by itself may induce the development of autistic symptoms [142]. Indeed, early-life seizures may affect synaptic plasticity through alteration of neurotransmitter receptor systems and molecules essential for intrinsic neuronal function, and changes that may contribute to an enhanced risk of autism [143,144]. Supporting this hypothesis, infantile spasms and/or early-onset seizures have been significantly associated with autism [145-147]. In the present study, few children had seizures in their first year of life (11/63, 17%) and none had infantile spasms. Thus, this mechanism may not account for much of the high prevalence found. As stated previously, in individuals with autism from multiplex families, common pathophysiological mechanisms appear to be more prevalent and may be genetic. If both autism and epilepsy can be considered as disorders of synaptic plasticity [143,144], autism and epilepsy may be the consequences of the same pathophysiological mechanisms, resulting in abnormal plasticity of genetic origin. Numerous genetic conditions that associate autism and epilepsy in their phenotypic features are caused by mutations in genes implicated in synaptic development, functioning and/or plasticity, such as Angelman syndrome [148], fragile X syndrome [149], Rett syndrome [150] and tuberous sclerosis [120,151]. Furthermore, missense and nonsense mutations of several genes involved in neurodevelopment have been identified in individuals with epilepsy, autism or both (Table 1). Most of those genes are involved in cerebral development pathways and functioning: synaptic formation (*NLGN4*, *NRXN1*, *CDH8*, *PCDH19*, *SHANK3*), ionic channel subunits (*SCN1A*, *SCN2A*, *SCN1B*, *KCNMA1*, *KCNQ2*, *CACNA1A*) and neurotransmitter receptors (*GABRG2*, *CHRNA4*). Similarly, CNVs such as 1q21.1, 3q29, 7q11.23, 15q11–13, 15q13.3, 16p11.2, 16p13.11, 17q12 and 22q11.2 have been implicated in different developmental disorders, such as ASD, intellectual disability and epilepsy [15,17,152], as well as schizophrenia [16,152,153] (Table 2). Autism, epilepsy and intellectual disability may share alteration of common pathways involved in neurodevelopmental maturation and neural functioning. We propose that these genes need to be more systematically investigated as major and minor risk factors.

Although the current study was conducted in the largest available sample of multiplex families, limitations should be listed. First, we cannot exclude biases in recruitment due to exclusion criteria as evidenced by the very low rate of infantile spasm in the sample. Furthermore, of the 664 children included in this analysis, 35 children (31 children with autistic disorder, two children with PDD-NOS and two children without autism) had strong risk factors for autism and seizures, such as pre- or perinatal insult/prematurity before 35 weeks (n = 19), chromosomal abnormality (n = 8), or abnormal neurological or brain imaging examination (n = 7). However, when analyses were performed excluding these children and their siblings, the results remained unchanged (see also Additional file 1). Second, individuals’ ages were significantly different in children with autism with epilepsy, and children with autism without epilepsy, and the small number of adolescents may have reduced the prevalence of late onset seizures. Third, given the heterogeneity of intellectual functioning in autism and the heterogeneity of intellectual disability measures, we used two standardized tools for the cognitive and adaptive assessment: RCPM and VABS. Assessing a broad sample of 38 autistic children, Dawson *et al*. found scores on the RCPM to be, on average, 30 percentile points higher than their scores on the Wechsler scale of intelligence [154]. The VABS assess the adaptive functioning with a parent interview. Strong positive relationships have been found between Vineland composite and Vineland subscales (communication, socialization, daily living skills) and IQ, particularly when considering low functioning individuals [155]. Therefore, the two scales can be considered as complementary for the cognitive assessment of children with autism. The fact that we found similar results in the AGRE sample strengthens this view. Fourth, we cannot exclude that the absence of association with gender was related to our final sample size and a lack of statistical power. To assess this issue, we decided to perform a meta-analysis in a larger sample including other studies reporting IQ in multiplex pedigrees [126,138]. The standardized mean difference between mean non-verbal IQ in males versus females from multiplex pedigrees was 0.01 (95% CI: –0.17; 0.19), meaning that there is no difference between mean non-verbal IQ according to gender (Additional file 2: Figure S1). The meta-analysis confirmed our results.

## Conclusion

We conclude that epilepsy in multiplex autism may define a different subgroup in terms of clinical characteristics and genetic risk: association with intellectual disability, no association with gender and possible common risk factors. Future studies should be encouraged to increase knowledge of common genetic risk factors between ASD and epilepsy.

## Abbreviations

aCGH: Array comparative genomic hybridization; ADI-R: Autism diagnostic interview-revised; AGRE: Autism genetic resource exchange; ASD: Autism spectrum disorders; CI: Confidence interval; CNV: Copy number variation; EEG: Electroencephalogram; GABA: γ-aminobutyric acid; GLUT1: Glucose transporter type 1; GWA: Genome-wide association; ID: Intellectual disability; IQ: Intellectual quotient; nACh: Nicotinic acetylcholine; NQA: Not quite autism; OR: Odds ratio; PCR: Polymerase chain reaction; PDD: Pervasive developmental disorder; PDD-NOS: Pervasive developmental disorder-not otherwise specified; RasGAP: Ras GTPase activating protein; RCPM: Raven’s colored progressive matrices; SCZ: Schizophrenia; SD: Standard deviation; SNRPN: Small nuclear ribonucleoprotein protein N; VABS: Vineland adaptive behavior scales.

## Competing interests

CA and BG are salaried employees of IntegraGen (Évry, France). DC and ST are compensated consultants for IntegraGen. IGA, CL, EL and NB declare that they have no competing interests.

## Authors’ contributions

CA, DC, CL, ST and EL designed the study. CA, BG and IGA extracted the data and performed the quality assessment. CA, IGA, EL and CL critically reviewed the literature. NB, BG, CA, CL and DC performed the statistical analysis. NB, CA and DC performed the meta-analysis. CA, CL, IGA and DC wrote a preliminary draft. All authors read, critically modified and approved the final manuscript.

## Supplementary Material

Additional file 1 Secondary analyses. Click here for file

Additional file 2: Figure S1 Standardized mean difference between mean non-verbal IQ in males versus females from multiplex pedigrees: a meta-analysis of three multiplex autism pedigrees (n = 719). Click here for file
