# Supplementary material for: Does epilepsy in multiplex autism pedigrees define a different subgroup in terms of clinical characteristics and genetic risk?
Source: Mol Autism. 2013 Dec 1;4:47. doi: 10.1186/2040-2392-4-47 (PMC4176303; doi:10.1186/2040-2392-4-47)
Supplement: Additional file 1 — Secondary analyses. [file 2040-2392-4-47-S1.doc]

**Supplementary 1**

As the AGRE database included also several cases that were flagged because of presence in the family of one subject with a risk factor of ASD (see method section), to assess whether inclusion of these families biased our results, we performed secondary analyses excluding all flagged cases. These analyses were performed on 586 children from 257 families who had informative medical data about presence or absence of epilepsy: 362 children with autistic disorder, 53 children with PDD-NOS and 171 children without autism. Overall, the results remained similar for all analyses performed except for association of epilepsy and Raven’s Colored Progressive Matrices score that show only a statistical tendency (p=.079, see below). Prevalence, type of epilepsy and clustering analysis are given in the main result section. Here we detailed the secondary analyses assessing whether higher prevalence of epilepsy in multiplex autism was mediated by intellectual disability (ID) and sex as generally reported in literature on autism.

*Epilepsy and non-verbal IQ/adaptive level*

Given the heterogeneity of general cognitive function in ASD, we used two measures to assess whether epilepsy was associated with ID in multiplex autism. An assessment with the Raven’s Colored Progressive Matrices was proposed to 383 children with ASD (334 children with an autistic disorder, 49 children with PDD-NOS). One hundred twenty four children were not testable and 9 children scored above the highest possible age-specific non verbal IQ, without numeric score informed. Mean non verbal IQ of children with ASD (N=250) was 100.2 (±17.6). Stratification of cases in two categories: IQ<70 (N=13) and IQ≥70 (N=246) showed a 4 fold increased risk for epilepsy for children with an IQ<70 (4/13; 30.8%) comparing children with IQ≥70 (24/246; 9.8%) (OR=4.1; 95%IC: 0.9-16; p=.079).

An assessment with the Vineland Adaptive Behaviors Scale was performed to 329 children with ASD (287 children with an autistic disorder, 42 with PDD-NOS). The mean overall Adaptive Behavior Composite standard score of children with ASD was 54.2 (±19.4). Stratification of cases in two categories: Composite Standard Score <70 (N=249) and Composite Standard Score ≥70 (N=80) showed a 6 fold increased risk for epilepsy for children with a Composite Standard Score <70 (35/249; 14.1%) comparing children with a Composite Standard Score ≥70 (2/80; 2.5%) (OR=6.4; 95%IC: 1.6-55.8; p=.004). Figure 1b reports the frequency of comorbid epilepsy in subjects with ASD as a function of. Moreover, the more severe the adaptive level (VABS Composite Standard Score) was impaired, the more prevalent was epilepsy (p=0).

*Epilepsy and sex*

Among the 85 females with a diagnosis of ASD (autistic disorder or PDD-NOS), 14 (16.5%) had an epilepsy. For the 330 males with ASD, 33 (10%) had an epilepsy. The male:female ratio was 4.2:1 for the children with ASD without epilepsy and 2.4:1 for the children with ASD and with epilepsy. However, the difference was not statistically significant (OR=0.6; 95%IC: 0.3-1.2; p=.145).

Considering all the children with ASD whatever their epileptic status, the male:female ratio was not significantly different when the sample was stratified in two groups (normal intelligence vs. ID) according to the non-verbal IQ (RCPM: ≥70, N=246; <70, N=13) (Male/Female ratio=4.5 vs. 3.3, respectively; OR=0.7; 95%IC: 0.2-4.4; p=.89) or the adaptive level (VABS: (≥70, N=80; <70, N=249) (Male/Female ratio=3.4 vs. 3.8; OR=1.1; 95%IC: 0.6-2.1; p=.869). Furthermore, there was no difference when the sample was stratified in four groups according to the adaptive level (<40, 40-54, 55-69, ≥70) (p=.699).
