# Supplementary material for: Does epilepsy in multiplex autism pedigrees define a different subgroup in terms of clinical characteristics and genetic risk?
Source: Mol Autism. 2013 Dec 1;4:47. doi: 10.1186/2040-2392-4-47 (PMC4176303; doi:10.1186/2040-2392-4-47)
Supplement: Additional file 2: Figure S1 — Standardized mean difference between mean non-verbal IQ in males versus females from multiplex pedigrees: a meta-analysis of three multiplex autism pedigrees (n = 719). [file 2040-2392-4-47-S2.doc]

In the present study, the respective prevalence rates of epilepsy was 41.2% in individuals with autism and ID, against 11.1% in individuals with autism and normal intelligence (p=.005) and the more the adaptive behavior level was impaired, the more epilepsy was prevalent. However, there was no increase of epilepsy associated with sex. Furthermore, no significant difference in IQ among males and females was found in the AGRE sample. A similar result has been already suggested in previous reports studying multiplex families [1-2]. As we cannot exclude that the absence of association with gender was related to our final sample size and a lack of statistical power, we decided to perform a meta-analysis to assess this issue in a larger sample.

A meta-analysis comparing males and females was performed using the R package “meta”. We extracted data relevant for non-verbal IQ per sex in three different studies: Spiker et al (2011) included 142 individuals with ASD from multiplex pedigrees and assessed non-verbal IQ using Leiter scale. Banach et al (2009) included 288 individuals with ASD from multiplex pedigrees and assessed non-verbal IQ using Leiter scale or WISC. In Banach study, data were available according to rank of siblings. Finally, we included the AGRE sample reported here with individuals that were assessed with Raven Non Verbal IQ (N=289). In total, 719 individuals (548 males, 171 females) were included in the meta-analysis. Since different scales were employed to measure IQ, and since this outcome is continuous, a standardized mean difference was used as measure of outcome effect. Results are given for both fixed effect and random effect models. The estimated variance between studies is 0 (DerSimonian-Laird estimator), thus there is no difference between the two models. Standardized mean difference between mean non verbal IQ in males vs. females from multiplex pedigrees was 0.01 (95%CI:-0.17; 0.19), meaning that there is no difference between mean non verbal IQ according to sex (figure S1).

**Figure S1. Standardized mean difference between mean non verbal IQ in males vs. females from multiplex pedigrees: a meta-analysis of 3 multiplex autism pedigrees (N=719)**

1. Spiker D, Lotspeich LJ, Dimiceli S, Szatmari P, Myers RM, Risch N: **Birth order effects on nonverbal IQ scores in autism multiplex families.** *J Autism Dev Disord* 2001, **31:**449-460.

2. Banach R, Thompson A, Szatmari P, Goldberg J, Tuff L, Zwaigenbaum L, Mahoney W: **Brief Report: Relationship between non-verbal IQ and gender in autism.** *J Autism Dev Disord* 2009, **39:**188-193.
